# Loss of safety in numbers and a novel driver of mass migration: radiotelemetry reveals heavy wasp predation on a band of Mormon crickets

**DOI:** 10.1098/rsos.160113

**Published:** 2016-05-04

**Authors:** Robert B. Srygley, Patrick D. Lorch

**Affiliations:** 1 Northern Plains Agricultural Research Laboratory, USDA-Agricultural Research Service, 1500 N. Central Avenue, Sidney, MT 59270, USA; 2 Biological Sciences Department, Kent State University, Kent, OH 44242, USA

**Keywords:** parasitism, digger wasp, numerical response, Orthoptera, katydid, biological control

## Abstract

Coordinated movement of animals is a spectacular phenomenon that has received much attention. Experimental studies of Mormon crickets and locust nymphs have demonstrated that collective motion can arise from cannibalism that compensates for nutritional deficiencies arising from group living. Grouping into migratory bands confers protection from predators. By radiotracking migrating, Mormon crickets released over 3 days, we found that specialized, parasitoid digger wasps (Sphecidae) respond numerically and prey heavily on aggregated Mormon crickets leading to loss of safety in numbers. *Palmodes laeviventris* paralysed and buried 42% of tagged females and 8% of the males on the final day of tracking. Risk of wasps and Mormon crickets hatching on the same site is high and may drive nymphal emigration. A preference to provision offspring with adult female Mormon crickets can be explained by their greater fat content and larger size compared with males, improving survival of wasps during diapause.

## 1. Introduction

Group living benefits animals in part by providing them with protection, whereas costs of living in groups often lead to collective movement. Being in groups confers protection from predators owing to safety in numbers [1,2], early detection [3] and predator confusion [4,5]. As group density increases, however, costs of group living increase as well owing to factors such as nutrient scarcity and cannibalism [6], increased disease transmission [7], and the functional and numerical responses of predators to prey density [8,9]. Some of the most spectacular animal mass movements, from gazelles to locusts, have been interpreted in this cost–benefit framework of protection from predation and cost reduction. This framework has been the focus of a number of theoretical and empirical studies. However, empirical studies have trailed theoretical ones because of the difficulty of tracking individuals in groups, and also because predation events and the causes of collective movement are difficult to observe. Advances in tracking [10] and the ability to capture, measure or manipulate phenotypes, and release individuals back into migratory bands have made Mormon crickets *Anabrus simplex* (Orthoptera: Tettigoniidae) and nymphal locusts (hoppers) model organisms for understanding collective motion in this cost–benefit framework [11].

Group living provides Mormon crickets with protection from some predators. To demonstrate this, Sword *et al.* [12] collected Mormon crickets from three bands and released some of them as solitary individuals 5 km away from their bands of origin, whereas controls were released back into their migratory bands. Mormon crickets were radiotracked for 2 days to measure survivorship. Although none of the control Mormon crickets were lost from within the bands, 50–60% of the solitary Mormon crickets were lost within 2 days. Predation on solitary individuals was evident from damage to putty surrounding the recovered radios and broken body parts that remained attached to the radios, although specific predators were not identified.

However, group living can also be detrimental. Grouped individuals are in more frequent contact and transmit disease more readily [13]. Grouping also increases competition for food leading to deficiencies in macronutrients, such as protein and carbohydrates. Compensating for nutritional deficiencies with cannibalism can force collective motion [6,11,14–16]. For example, Mormon crickets with protein and salt deficiencies cannibalize one another [6]. Hence, a prevailing view of the migratory band is a push-me pull-you tension with Mormon crickets running ahead of those behind to avoid being cannibalized and those behind running to keep up with the leaders to avoid becoming solitary and at greater risk of predation. In addition, insects that divert from the common direction of the band increase the contact frequency and risk of cannibalism by neighbouring band members [16]. Mormon crickets at the lateral limits of the band have a similar conflict: movement away from the band reduces risk of cannibalism but increases predation risk, whereas movement into the band reduces predation risk while increasing intraspecific encounters and the risk of cannibalism. More recent research indicates that some bands are composed of Mormon crickets that are carbohydrate deficient [17,18]. Although cannibalism of dead or wounded individuals occurs (R.B.S., personal observations) and migration slows after encountering carbohydrates [17,18], what drives collective motion in these carbohydrate-seeking Mormon cricket bands is unknown.

Two species of parasitic digger wasps (*Palmodes laeviventris* and *P. hesperus*, Sphecidae) specialize on Mormon crickets to provision their offspring [19]. Additional wasps (e.g. *Stizoides unicinctus*) and flies (e.g. *Eumacronychia elita*) exploit the activities of the digger wasps [20]. Hence, Mormon crickets must be a sufficiently reliable food source for parasitoids, and hyperparasitoids, to evolve specialized behaviours to exploit them. Despite the ecological and economic importance of mass migrating Mormon crickets on rangeland in the western US [21], predation by parasitoid wasps on Mormon crickets has only rarely been investigated [20,22]. By radiotracking Mormon crickets in a migrating band, we measured predation by parasitic wasps that gives insight into novel costs of group living and suggests a driver of collective motion that was not previously recognized.

## 2. Material and methods

Mormon crickets are flightless katydids that form dense aggregations that often move like bands in a single direction. Bands can be 1–2 km in width and tens of kilometres in length [21]. Although little is known about band formation, it can occur when Mormon crickets have moulted to fourth-instar nymphs or later. Mormon crickets typically march in bands in the mornings and evenings, climb vegetation to feed in late morning, and often rest in vegetation above the ground or enter burrows and cracks in the ground to evade midday heat at ground level (R.B.S., personal observations).

**Figure 1.**
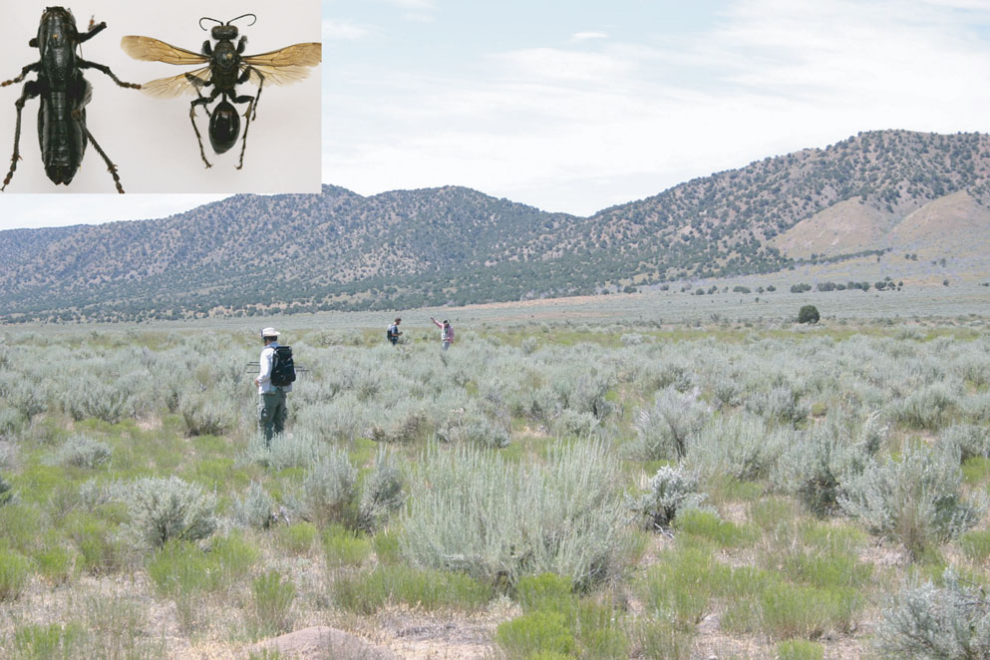
Radiotracking Mormon crickets on the study site in Furnar Valley, Utah. Inset: male Mormon cricket and female *P. laeviventris* digger wasp. Body length for the Mormon cricket is approximately 4 cm and that for the digger wasp 3 cm.

We studied a band of adult Mormon crickets in Furnar Valley, Utah (figure 1, latitude 39°43′37′′ N, longitude 112°4′34′′ W, 1780 m elevation) between 14 and 17 July 2011. The floor of the valley is dominated by sagebrush (*Artemisia tridentata*), typical vegetation for high Great Basin desert scrubland.

In order to follow the migratory path of each Mormon cricket, we used standard methods to radiotrack Mormon crickets without affecting their locomotion and other behaviours [23,24]. An equal number of male and female Mormon crickets were collected from the band, and a 0.4 g radiotransmitter (Biotrack, Ltd, UK) was glued to the pronotum. We recorded the Mormon cricket's sex and the unique radiofrequency that identified each insect, and then returned the insects to the band with each Mormon cricket's location and time of release recorded with a Trimble Geo XT GPS device (accuracy ± 30 cm). At release, each cricket was separated from neighbouring marked individuals by *ca* 8 m on a linear transect perpendicular to the general direction of the movement of the band at that time. Mormon crickets were released between 17.25 and 18.00 h and radiotracked the following day to the site of the radio, typically with the insect still attached, between 11.00 and 13.40 h. The Mormon cricket's (or radio's) location and time of recapture were recorded with Trimble GPS dataloggers. An examination of radios for gnawing or other signs of predation was used to separate those that had become accidentally unglued. Some radiotagged Mormon crickets were dug from burrows in the soil, and these were inspected for parasitoid wasp eggs and paralysis. Paralysed Mormon crickets and those with attached wasp eggs or larvae were scored as prey. The methods were repeated on a new set of Mormon crickets over three successive 24 h periods (*n* = 15 females and 15 males on 14 July, *n* = 14 females and 14 males on 15 July and *n* = 14 females and 14 males on 16 July). Prior to the first release, the Mormon crickets were weighed, but for subsequent releases, only recaptured Mormon crickets were weighed. For those released on 14 July, one male and one female were not recovered, probably because their radios' batteries died, and one female lost its radio. For those released on 15 July, two females were not recovered and one male and one female lost their radios; and for those released on 16 July, two females were not recovered, two males lost their radios. Unrecovered Mormon crickets, including those with lost radios, were excluded from the predation counts (figure 1) and velocity measurements. On both 15 and 16 July, the recapture point for one insect was mistakenly not logged. Although their velocity could not be measured, these Mormon crickets are included in the predation counts. Velocity of those still capable of moving was calculated as the straight-line path between the points of release and recapture divided by the time between the events. Velocity was log-transformed to normalize the data (Shapiro–Wilk goodness of fit test: *W* = 0.97, *p* = 0.119).

We tested whether one sex was more susceptible to predation with Pearson's chi-squared analysis; and we tested whether body mass was a predictor of predation risk with Goodman's log-likelihood analysis. We also investigated whether parasitoid wasps predated selectively on slower individuals with a two-way analysis of variance of log velocity by sex and date. All statistical tests were performed using JMP v. 9 (SAS Inc.).

## 3. Results

**Figure 2.**
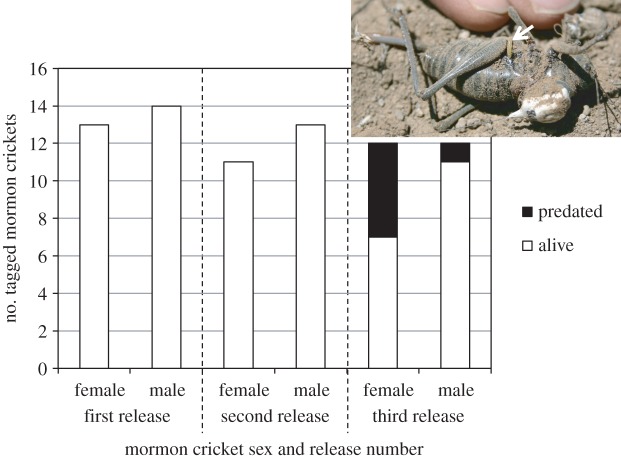
Number of radiotagged male and female Mormon crickets that were predated by digger wasps (*P. laeviventris*) during each of three successive evenings of release and recapture the following day. Inset: a radiotagged Mormon cricket female that was recovered from a wasp burrow. The white arrow points to a wasp egg at the base of the Mormon cricket's hindleg.

Predation rate changed suddenly from one night to the next (figure 2). Although none of the radiotracked Mormon crickets were predated on the first two nights, 25% were predated on the third night (five females and one male).

**Figure 3.**
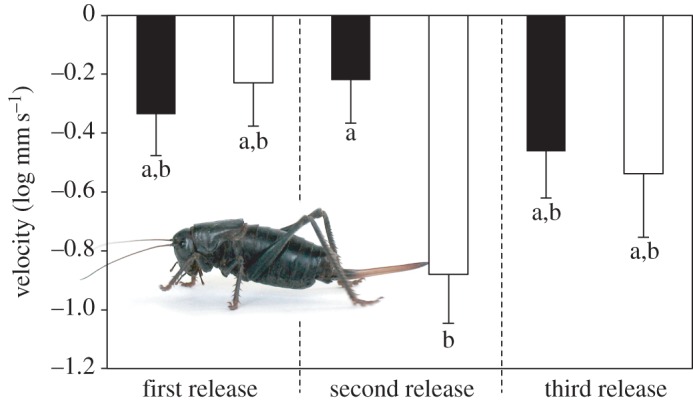
Velocities of male (solid bars) and female (open bars) Mormon crickets between release and recapture over three nights. Females were significantly slower than males on only the second release, whereas the wasp predation occurred following the third release. A female Mormon cricket distinguished from the male by the long ovipositor is illustrated.

Females were predated more than males (figure 2, Pearson's chi-squared test: *χ*^2^ = 4.39, d.f. = 1, *p* = 0.037). From our relative efficiencies of capturing the two sexes for tagging, there was no evidence that the band was biased from an even sex ratio. Body mass was not a good predictor of predation risk (log-likelihood *G* = 0.084, d.f. = 1, *p* = 0.68). Log velocity did not differ between those males and females that survived during the last 24 h period (*F*_1,15_ = 0.133, *p* = 0.133), but owing to the exclusion of predated Mormon crickets, the sample size was low, particularly for females. Including all three dates, the interaction of sex and date released had a significant effect on log velocity (model *R*^2^ = 0.16, *F*_5,60_ = 2.34, *p* = 0.052; interaction *F*_2,60_ = 3.54, *p* = 0.035), with females significantly slower than males on only the second release (figure 3).

## 4. Discussion

Predation on Mormon crickets released into the band was heavy and sex-biased. The loss of 25% in 1 day within the band was very similar to that measured on Mormon crickets released alone outside the band (25–30% per day, [12]). We identified the predator as the sphecid wasp *P. laeviventris* (Cresson, figure 1), which is only known to predate on Mormon crickets [19]. Mormon crickets with radios attached were recovered from burrows of *P. laeviventris*, in which they lay paralysed with a single wasp egg laid on their sides. On the final day of tracking, *P. laeviventris* paralysed and buried 42% of tagged females and 8% of the males. All open burrows in the area were newly dug from above, making it evident that the wasps had not emerged from within the vicinity of the band.

Instead, the wasps appeared to have responded to the band numerically with the wasps moving into the area during the tracking period. After our study ended on 17 July, seven female wasps, distinguishable from males by their large body size, were observed flying over the area between 16.30 and 17.00 h, apparently scanning for Mormon crickets. LaRivers [20] also notes a numerical response by the wasps to a band of crickets on 13–20 July 1939 in the Bull Run Mountains near Elko, Nevada: ‘cricket invasions of the higher, western end of the ridge brought a new influx of wasps’. He estimates that 30 000 wasps worked the 320 acres (129 ha) that a migratory band covered. In one acre (0.4 ha) where densities of wasps and Mormon crickets were greatest, LaRivers [20] found an average of six completed burrows per square yard by the end of 6 days after the initial appearance of the Mormon crickets and wasps. From this, he estimated that the wasps buried 58 000 crickets in that one acre in 6 days. Note that at 20 Mormon crickets per m^2^, a reasonable estimate for a band at high density [25], the wasps would have parasitized 72% of the 80 000 crickets on the acre. From quadrats sampling the 129 ha area of the band, he estimates a total of 256 500 burrows containing 513 000 paralysed crickets were constructed in the same period.

Hence, at the initial loss of 25% of the Mormon crickets per day that we measured, *P. laeviventris* has the potential to make short work of a band of Mormon crickets. This represents a loss of safety in numbers [12], because a burrow-constructing specialist like *P. laeviventrus* is able to provision more offspring as the distance it needs to drag Mormon crickets to its burrow decreases. LaRivers [20] found that the average distance from prey capture to the nest was 7 m, and the wasp showed difficulty finding the nest if the distance was greater than 12 m. In addition, the likelihoods of other wasps contesting prey ownership or parasitic flies encountering the wasp and its prey increase as the distance (and time) to the burrow increases. Hence, the wasps should respond positively to Mormon cricket density.

A specialized foe, such as *P. laeviventrus*, is a sufficient selective force to propel migration of Mormon crickets. Indeed, theoretical results suggest that migration is necessary for both host and parasitoid species to persist [26,27]. Because the wasps provision their offspring with adult Mormon crickets, the wasps' burrows will often lie in areas where Mormon crickets have laid eggs. In the following summer, adult wasps emerge in the same sites where Mormon crickets have hatched the previous spring. This increased risk of predation on natal sites should strongly favour emigration of Mormon cricket nymphs, even before the wasps have emerged. Safety from other predators then binds Mormon crickets in aggregation, and cannibalism contributes to the collective motion of some bands on a forced march for protein and salt [6]. Selection to avoid digger wasp predation explains why Mormon crickets that are carbohydrate-limited migrate, even though cannibalism may be less pronounced [17,18]. Predation by other digger wasp species on locusts (e.g. *Sphex aegyptius* on the desert locust *Schistocerca gregaria* [28,29]; *Sp. cognatus* on the Australian plague locust *Chortoicetes terminifera* [30]) may also be a driver for these orthopterans to migrate from natal grounds. Although *Sc. gregaria* adults fly faster than either locust nymphs [28] or Mormon cricket adults walk, *Sp. aegyptius* tracks locust swarms, burrowing and paralysing adult locusts to provision their young during stopovers [28].

*Palmodes laeviventris* emerges after Mormon crickets have reached adulthood which allows it to provision its offspring with Mormon crickets that are much larger than immature stages. As a result, bands are predated by wasps long after Mormon crickets band together in their nymphal stages. Therefore, banding still confers protection from predators for much of the Mormon cricket's lifespan. Moreover, if we assume that wasp populations crash when Mormon cricket densities decline, then a lag of several years between the expansion of Mormon cricket populations and those of the wasp probably provides additional protection from wasp predation.

Preferential predation on females greatly enhances the negative effect of *P. laeviventris* on Mormon cricket reproduction. In six wasp nests, we found six females to one male. Gwynne & Dodson [22] found 30 females to 4 males in 19 wasp burrows. As a result, the number of eggs laid would decline twice as fast as when predators are unbiased in their selection of the sexes.

Several hypotheses to explain the wasps' female bias in provisioning offspring have been proposed. Gwynne & Dodson [22] postulated that the wasps prefer females because they are larger. In their study, masses of provisioned insects were contrary to the hypothesis, although the number of males was too few for a statistical test. In our study, we too did not find evidence that the wasps' preference for females was owing to a difference in body mass. However, in the laboratory on ad libitum diets, adult females and males are the same mass after moulting, but females gain mass faster than males (160 versus 95 mg day^−1^, [31]). As a result, mass differences in the field might depend on adult age, and wasps could have evolved a preference for females because they are typically heavier. The geometrical mean of ten measurements (including volume, three head size variables, three pronotum size variables and three leg size variables) was significantly larger in females than males collected in the field (P.D.L. 2008, unpublished data). Mass differences also appear to be dependent on age in another field study where adult females were larger than males with dry weights 40% heavier shortly after moulting and 100% heavier later in the season [25].

A second hypothesis proposed by Gwynne & Dodson [22] is that sex role reversal in banding Mormon crickets, in which males prefer larger females and females compete for access to nuptial gifts [32], makes females more vulnerable to predation. Sex-biased predation on the less-limiting prey sex (e.g. females in sex-role reversed Mormon crickets) can stabilize predator–prey dynamics [33]. Typically, the calling sex is more vulnerable to predation [34,35], and even with males becoming the choosier sex in Mormon cricket bands, they remain the displaying sex [32]. Female choosiness causes females to move more to find calling males and fight for access to them. As suggested by Gwynne & Dodson [22], this difference in movement could be the source of increased vulnerability of female Mormon crickets to predation. However, in *Poecilimon* bushcrickets, survivorship of males and females is not different when one sex displays and the other moves towards the signal [36]. In contrast, if one sex displays, the other responds discretely, and then the first moves towards the respondent, mortality was much greater in the moving sex. Because the former scenario is more like that for Mormon crickets, predation should be even between the sexes. Moreover, in the Australian plague locusts *C. terminifera*, digger wasps prefer males and females in copula to solitary individuals [30]. Even in this system where the wasp is given a free choice between the sexes, it chooses to paralyse the female to provision the offspring with the unfortunate male often buried unparalysed in copula with its mate.

We propose an alternative hypothesis that the sexes are nutritionally different. Using published data [25], we examined whether Mormon cricket sexes differ in protein or lipid composition. Protein composition is slightly higher in male Mormon crickets (60% dry weight versus 56%), but lipid content is much higher in females (20% dry weight versus 13%, [25]). In many insects, access to fat reserves prolongs survival in diapause, particularly at higher temperatures [37]. The emergence of adult wasps from their burrows in July suggests that they spend several months of the spring and summer underground at temperatures where they are quickly using energy resources without being able to acquire them. Hence, survival of the offspring during diapause might select for a maternal preference for female Mormon crickets with higher fat content than males.

Digger wasps sometimes take Mormon crickets that are infected with endoparasitic *Sarcophaga* fly larvae (9 of 211 crickets that LaRivers [20] exhumed were infected by this fly), which suggests that the wasps might prefer to attack and capture weakened, and hence slower, Mormon crickets. We found that females were slower than males only after the second release during which wasp predation was not evident. However, during the release when the wasp predation occurred, female survivors were not slower than males. In the tree cricket (*Oecanthus nigricornis*), jumping ability declined and predation by sphecid wasps increased with egg load [38]. Greater predation on egg-laden females also supports the hypothesis that the wasps prefer the more nutritious, lipid-rich sex, but we cannot exclude the possibility that the wasps' preference for female Mormon crickets could also arise from movements, such as jumping ability, that we did not measure by radiotracking.

Even if females are temporarily slower than males, the selfish herd hypothesis [1] predicts that the predatory bias to capture females and the limited range in which the wasp can range from its burrow to find prey will select for male Mormon crickets that remain near females and provide the wasps with the opportunity to select the preferred sex. Thus, males should do their best to match the velocity of females when wasps are present.

## 5. Conclusion

Numerical response to Mormon cricket bands coupled with a preference for females to provision their offspring make the digger wasps particularly effective at controlling Mormon cricket populations. Wasps may play an important role in causing density declines leading to periods where Mormon crickets are only found sporadically throughout their range. Wasp predation represents a cost to collective movement resulting in a loss of safety in numbers. It also suggests that avoidance of this specialized predator should strongly select for Mormon cricket emigration from natal sites, adding to the threat of cannibalism as a selective force spurring collective motion.

## Supplementary Material

Radio tracking data including set down and pick up GPS locations for each insect

## Data accessibility

The dataset supporting this article has been uploaded as electronic supplementary material.
